# When the AI Self Becomes the Ideal Self: A Systematic Review of AI-Enhanced Self-Discrepancy, Appearance Anxiety, and Protective Psychological Factors

**DOI:** 10.3390/bs16101822

**Published:** 2026-10-05

**Authors:** Anju Soman, Navneet Mishra, Sumona Chakladar

**Affiliations:** 1 Department of Psychology, Alliance University, Bangalore 562106, Karnataka, India; 2 Department of Psychology, Faculty of Psychology, Alliance University, Bangalore 562106, Karnataka, India; 3 Department of Business, Alliance University, Bangalore 562106, Karnataka, India

**Keywords:** artificial intelligence, AI beauty filters, self-discrepancy, appearance anxiety, body image, positive body image, social comparison, self-compassion and protective factors

## Abstract

Artificial intelligence (AI) appearance technologies, including beauty filters, augmented reality (AR) modification and generative avatars, have individualized digital self-presentation. Unlike traditional media, these technologies create self-referential appearance ideals that create novel forms of self-discrepancy and appearance anxiety. A systematic review was conducted in peer-reviewed databases for empirical studies published from 2021 to 2026 that studied self-representation, self-discrepancy, appearance distress, and protective psychological constructs within this context of AI, following PRISMA 2020 guidelines. Emergent patterns were identified by using the narrative thematic synthesis method across eligible studies. A total of eight empirical studies were found that fulfilled the inclusion criteria. The themes that emerged were as follows: (1) AI-enhanced self as a novel appearance ideal of the self; (2) self-discrepancy as an operative mechanism between the appearance of idealized and non-idealized selves and psychological distress; (3) contextually related and protective moderators; and (4) representation modality—between aestheticizing versus non-aestheticizing appearance modalities and psychological distress. The findings from the synthesis showed that self-discrepancy, social self-comparison, and self-objectification are the three main routes for appearance anxiety. Theoretically, these non-idealizing avatars, high self-similarity, and goal-oriented use contexts were considered as protective buffers but were not consistently quantified. Most importantly, there was no empirical study that directly assessed positive body image constructs or body appreciation. The current research mainly focuses on the pathology aspect of AI appearance technology, and positive body image is not investigated. Empirical studies should be conducted in the future with the clear aim of examining if constructive, nonidealized relationships with the technologically enhanced body can foster psychological resilience, and if so, how.

## 1. Introduction

The concept of artificial intelligence (AI) has transformed people’s lives through a great revolution, which has changed their interaction, fashion and representation in the virtual world. With the help of beauty filters, augmented reality (AR) apps, facial enhancement tools, and avatar generation technologies, users can create highly optimized, aesthetically enhanced, and beautified versions of themselves using AI technology with ease. Modern AI tools can automatically manipulate facial symmetry, skin texture, body shape, and other facial characteristics that influence appearance, making them more similar to cultural beauty standards, unlike previous photo editing technologies (Ozimek et al., 2023). In the social media space, these technologies are now so part of the fabric of the world that self-presentations made via these media are now the norm and are being widely shared. This has resulted in a murky area between real and augmented appearance and raised serious questions about the psychological consequences of AI’s self-representation.

In this review, AI-enhanced self-representation relates to any digital representation of an individual’s own appearance that is generated, modified, or rendered by an underlying computational or machine-learning process and that an individual can see as a representation of himself. This definition covers four types of AR technologies that were identified in the literature included: (1) AI beauty filters, which are used to detect a face in a static photo and apply generative image modifications (e.g., skin smoothing, facial reshaping) based on the use of machine-learning techniques; (2) AR real-time filters, which use computer-vision tracking to apply comparable transformations to a live video stream; (3) self-similar virtual humans, which are three-dimensional avatars created from a scan of the user’s body and can be manipulated for changes to their body weight or shape; and (4) user-configured avatars, which are non-photorealistic representations such as Memoji, which track the use’s movements or expressions but are built from a limited set of user-selected features rather than based on the user’s actual image. The common psychological property of these subtypes that is relevant to this review is that they are all representations that represent the user as if they are his or her own appearance, but not other people’s. They differ greatly in terms of modality (2D image manipulation versus 3D rendering); to what degree they are algorithmically transformed from the users’ actual features; and whether their representation is idealizing versus neutral or user-controlled by design. These are considered a unified category for the purposes of this review, and results are reported individually by sub-type where the main studies will allow (see Section 3.1.3).

The high rates of digitally manipulated self-presentations are representative of the rise in society’s emphasis on the visual aspects of self-presentation and identity construction in online environments. The perception of appearance, social validation and self-presentation are considered important elements of social interaction in social network platforms (Valkenburg et al., 2022). Many research studies have identified associations between viewing idealized appearance content online and body dissatisfaction, appearance concerns and negative body evaluations (Fardouly & Vartanian, 2016; Holland & Tiggemann, 2016; Saiphoo & Vahedi, 2019). The effects on these are typically explored with edited images of others, but the new psychological phenomenon is that people are now more exposed than ever to idealized images of themselves through technologies powered by AI. This shift may revolutionize the way people think about images of themselves, as AI-created images of self become images of self-standards.

The psychological meaning of this phenomenon can be explained by Self-Discrepancy Theory (Higgins, 1987), which suggests that psychological distress occurs when one’s self-perception of discrepancy between one’s actual self and one’s ideal self. The actual self is the set of attributes that people feel they possess, and the ideal self is the set of attributes that people would like to possess. The greater the discrepancy between actual and ideal, the more negative effects, less self-esteem and anxiety-related outcomes are related (Higgins, 1987; Schlechter et al., 2022). In the past, ideal selves were shaped by interpersonal expectations, media images and social norms. From this point, however, AI-generated images can create a new “ideal self” by generating personalized images that are visually idealized, realistically rendered, and feel attainable to the self. AI-generated images of one’s own body are built from one’s body image, as opposed to celebrity photos or media norms, and may therefore have a greater impact on their body image and sense of self.

Repeatedly seeing beauty filters, AI-generated avatars, and digitally manipulated self-portraits can exacerbate real/ideal body image gaps, according to new research. Body dissatisfaction, appearance dissatisfaction and body appearance modification needs are associated with filter exposure (Carter & Vartanian, 2022; Fioravanti et al., 2022). With repeated exposure to their idealized digital image, this can lead to a phenomenon known as “filter dysmorphia” (Ramphul & Mejias, 2018; Sadati & Radanfar, 2023), in which individuals may feel discontent with their actual appearance. The more advanced AI technology, the more the real and digitally created appearance could differ, which could further expose people to distress over their appearance.

Social comparison comes into play in the link between idealized digital self-representations and psychological well-being. According to social comparison theory (Festinger, 1954), people compare themselves with other persons’ characteristics to evaluate themselves. In such modern social media contexts, upward appearance comparisons are often frequent and have been linked to increased body dissatisfaction, appearance anxiety, and decreased self-esteem (Cohen & Blaszczynski, 2015; Fardouly & Vartanian, 2015). The comparisons can also work inward: when the AI-generated self-image makes a person think about themselves and their image, they might compare their physical self with the idealized version they created. The comparison target will likely be personally relevant, hopefully attainable, and such comparisons may be particularly compelling. In this way, the ideal self created by artificial intelligence can also be a very salient comparison standard that can continue to convey a sense of dissatisfaction with the physical self.

In addition to social comparison, objectification theory (Fredrickson & Roberts, 1997) provides a helpful framework to understand the role that AI-based appearance technologies can play in appearance anxiety. Objectification theory suggests that people absorb an observer’s perception of their body and adapt to the practice of viewing and observing one’s body. Self-objectification has been found to be associated with body shame, appearance anxiety, disordered eating and poor mental health (Kilpela et al., 2019; Schaefer et al., 2018). These are often compounded by the adoption of digital appearance modification technologies that may result in heightened awareness of self, self-evaluation and self-modification of appearance to fit within an ideal appearance. Thus, self-representations that are enhanced by AI can lead to more appearance monitoring and boost the self-objectification processes, which can raise appearance-related distress. Today’s digital environment has created appearance anxiety as an important psychological consequence. Social appearance anxiety is the anxiety resulting from negative evaluation based on physical appearance, and has been linked to several negative psychological outcomes, including low self-esteem, depressive symptoms, social withdrawal and lowered quality of life (Hart et al., 2008; Levinson & Rodebaugh, 2011). The number of appearance standards and self-presentation activities that are digitally mediated through social media sites has grown, which has impacted appearance anxiety (Jiotsa et al., 2021). But it is still unclear how these AI-generated ideal selves result in appearance anxiety. Not everyone who makes use of AI to look their finest will experience a negative psychological response.

*Positive body image as a separate protective factor:*

Although work on appearance technologies has been largely negative in terms of body-image outcomes (e.g., body dissatisfaction, appearance anxiety, self-objectification), these studies have not captured the positive aspects of appearance technology use. In the modern concept of body image, however, positive body image (PBI) is a unique and complex phenomenon, rather than simply the absence of these problems (Tylka & Wood-Barcalow, 2015b). PBI involves body appreciation, body acceptance and love (a holistic view of beauty), adaptive appearance investment, inner positivity (positive thinking about the body stemming from positive body regard), and protective filtering (selectively processing appearance-related information such that body-threatening information is rejected or reinterpreted instead of internalized) (Tylka & Wood-Barcalow, 2015b; Webb et al., 2015). Empirically, body appreciation has been shown to be only moderately negatively correlated with body dissatisfaction and accounts for unique variance in psychological well-being beyond negative body-image indices, which suggests that body appreciation is related but not redundant to body dissatisfaction (Tylka & Wood-Barcalow, 2015a; Avalos et al., 2005).

The difference has direct implications for the research into AI-enriched self-representation. PBI proposes a series of dispositions that can block pathways associated with idealized self-imagery at various stages, while self-discrepancy, social comparison and objectification theory do so at a single point. In particular, protective filtering refers to the exact opposite way in which an individual might come across an AI-generated self-image but not use it as a self-relevant ideal; that is, the idealized image is not even used as a self-relevant ideal in the first place. Likewise, a general definition of beauty could diminish the evaluative value of the beauty filters’ narrow, algorithmically driven features (smoothing skin, facial symmetry and facial slimming), and the appreciation of body and investment in appearance could separate global self-worth from real–ideal appearance congruence. Previous research has found that higher levels of body appreciation are related to less negative body image response to exposure to idealized body images in media (Halliwell, 2013), and that body appreciation is a protective factor for social media use and negative body image in general (Andrew et al., 2016; Cohen et al., 2019).

Positive body image is, as such, conceptually different from the overall psychological resources investigated in this literature. While self-compassion is always related to body appreciation, self-compassion and body appreciation are distinct and not synonymous, and self-compassion is a domain-general capacity that can moderate distress due to appearance concerns and other types of negative self-processing (Homan & Tylka, 2015; Neff, 2003). In much of this literature, self-esteem and appearance self-esteem serve as vulnerability moderators, yet also as protective resources. Lumping these together as an undefined “protective factor” ignores where each protective factor sits within the proposed pathway. Therefore, a classification of protective constructs is presented in the present review into four conceptually distinct groups: (i) body-image specific positive constructs (positive body image, body appreciation, body acceptance and functionality appreciation); (ii) domain-general self-related resources (self-compassion, resilience, self-esteem); (iii) regulatory processes (adaptive emotion regulation, cognitive reappraisal); and (iv) contextual and technological characteristics (representation type, use motivation, task framing). This taxonomy is used consistently in the synthesis (Section 3.1.4). With the knowledge of those protective processes and their place in action, theoretically supported interventions can be developed to enhance psychological health in the ever more AI-dominated social environment.

### Research Gap

The use of social media, appearance-related content, and exposure to digitally altered images have consistently been correlated with body dissatisfaction, self-objectification, and appearance anxiety. AI appearance technologies, on the other hand, add a unique layer of configuration: instead of rendering the idealized appearance of another person, they render the idealized appearance of the user, which can be produced, edited, and displayed repeatedly. The psychological correlates of AI beauty filters, AR filters, avatars, and appearance-enhancement applications have started to be documented, but there is a lack of a cohesive body of research, and individual studies tend to focus on one specific aspect of the phenomenon, such as body dissatisfaction, social comparison, self-objectification, or appearance anxiety.

Three gaps follow. First, there is not a lot of research investigating whether the AI-enhanced self-representation is most likely conceptualized as a self-referential self of the ideal self and whether discrepancies between the actual and AI-enhanced self are linked to appearance-related distress. Second, protective psychological constructs have been explored only occasionally in this context, and positive body image in particular, although one of the most directly implicated constructs by the notion of “appearance filtering”, has received little attention in research on the representation of the self via AI. Protective constructs that have been studied have typically been defined as a homogeneous group, with no differentiation made between body-image-specific constructs and resources, regulation mechanisms or contextual elements. Third, there is no systematic review that has synthesized evidence related to AI self-representation, self-discrepancy processes, appearance-related outcomes, and the entire spectrum of potentially protective constructs (such as positive body image). The gaps are relevant both in the theoretical development of the body-image models and in the identification of targets of interventions in an increasingly AI-mediated visual environment.

The present systematic review aims to condense and synthesize the current empirical literature addressing the role of AI-related self-representations and self-discrepancy, appearance-related outcomes, and protective psychological factors. The review aims to explore the connection between AI-generated and digitally enhanced self-representations and the development of self-referential appearance ideals; describe the evidence linking social comparison and self-objectification processes to the link between AI-enhanced self-discrepancy and appearance anxiety; identify and conceptually differentiate protective psychological constructs that have been reported in this body of literature, including positive body image and body appreciation, as well as domain-general resources, regulatory processes, and contextual characteristics; and evaluate the methodological soundness of available empirical evidence, and adjust theoretical inferences based on that assessment. The review seeks to outline how the use of AI for one’s own representation can be conceptualized as a means of either the ideal self or the negative self, and what this could suggest with respect to psychological functioning.

## 2. Method

### 2.1. Review Design

The methodology used to synthesize the empirical evidence for the relationships between AI-enhanced self-representations, self-discrepancy and appearance anxiety and protective psychological factors was a systematic review methodology. Methodological transparency, rigor and reproducibility (Page et al., 2021) were followed in conducting the review. This review did not have to be registered. Given the variability of study designs, populations, and outcome measures, and the emergence of findings in the literature, a narrative thematic synthesis approach was used to synthesize findings across studies. The review was conducted using the following theories: self-discrepancy theory (Higgins, 1987), social comparison theory (Festinger, 1954), and objectification theory (Fredrickson & Roberts, 1997); in addition, the positive body image framework (Tylka & Wood-Barcalow, 2015b) discussed in Section 1 was utilized. These theoretical frameworks were utilized for the analysis of the findings, and the conceptual framework of self-representations in an AI-driven era was employed to discuss the role of self-representation in appearance anxiety and protective psychological processes (self-discrepancy, social comparison, self-objectification, positive body image processes).

### 2.2. Sources of Information and Search Strategy

The search was carried out in the electronic databases Scopus and APA PsycInfo, which were chosen to provide the interdisciplinary scope of the review across psychology, body image research, communication and media studies, and human–computer interaction, with APA PsycInfo selected specifically to provide full coverage of the psychological literature, which Scopus has more extensive but shallower coverage of in terms of discipline-specific indexing. Scopus was searched by the first author (A.S.) in June 2026; APA PsycInfo was searched by the third author (S.C.) in September 2026, for records published between January 2021 and May 2026. The third author identified and evaluated records retrieved from the database and provided them to the first author, who screened them following the identical procedure described in Section 2.4. The two databases were searched on different dates because APA PsycInfo access was arranged after the initial Scopus search had already been conducted; both searches used the identical search string and date filters.

Search terms were grouped into five concept blocks and used in conjunction with the Boolean operators and truncation: (1) AI-enhanced and computationally mediated self-representation technologies; (2) self-discrepancy and ideal-self constructs; (3) negative appearance-related outcomes (body dissatisfaction, appearance anxiety, self-objectification); (4) positive body image constructs (body appreciation, protective filtering); and (5) protective and domain-general psychological resources (self-compassion, resilience, emotion regulation). The complete search string used in Scopus is presented below; the equivalent search string used in APA PsycInfo is presented in Supplementary Table S1.

TITLE-ABS-KEY((“artificial intelligence” OR “AI beauty filter*” OR “AI-generated image*” OR “augmented reality filter*” OR “AR filter*” OR avatar* OR “digital self” OR “virtual self” OR “virtual human*” OR “self-representation*”) AND (“self-discrepancy” OR “ideal self” OR “self-concept” OR “actual-ideal” OR “self-comparison”) AND ((“appearance anxiety” OR “social appearance anxiety” OR “body dissatisfaction” OR “body image” OR “self-objectification” OR “body surveillance” OR “appearance evaluation”) OR (“positive body image” OR “body appreciation” OR “body acceptance” OR “functionality appreciation” OR “protective filtering”) OR (“self-compassion” OR resilience OR “emotion regulation” OR “protective factor*” OR “self-esteem”))) AND PUBYEAR > 2020 AND PUBYEAR < 2027 AND (LIMIT-TO(DOCTYPE,”ar”) OR LIMIT-TO(DOCTYPE,”cp”)) AND (LIMIT-TO(LANGUAGE,”English”)). The asterisk (*) was used as a truncation/wildcard symbol to retrieve variations of the search terms.

There were 428 records in the Scopus search and 196 records in the APA PsycInfo search, which, before deduplication, totaled 624 records. Of the 196 records identified in APA PsycInfo, the majority overlapped with the Scopus results, consistent with substantial indexing overlap between the two databases for psychology and body-image journals. Only peer-reviewed journal articles or full papers in archival, peer-reviewed conference proceedings (as specified in Section 2.3) in English were accepted. Both databases were exported to one reference-management program, and 107 duplicate records (including cross-database duplicate records) were identified and removed before screening.

### 2.3. Eligibility Criteria

Studies were included if they explored some aspect of AI-based appearance technology or digitally altered self-representations, and if they reported results that were related to self-discrepancy, body image (negative or positive), appearance anxiety, social comparison, self-objectification or protective psychological factors. Eligibility criteria were developed before the screening process to ensure consistency and consistency with the aims of the review. The variables that were derived from each eligible study and the description of each variable are summarized in Table 1.

In addition to journal articles, full papers for presentation at archival conference proceedings submitted in a peer-review process were acceptable. The interdisciplinary nature of the review is reflected in this decision: while the proceedings of archival conferences are widely recognized as a central and highly refereed publication venue in the field of human–computer interaction, they are not considered a preliminary publication venue (allowing for the systematic exclusion of relevant evidence on avatar- and virtual-human-mediated self-representation). Conference abstracts and extended abstracts that were not archived were not eligible.

The intent of the technology context criterion (Table 1) was to be broad, which is why AI-assisted modification, AR filters, and computationally generated representations of oneself were grouped together as an eligibility category, and so were avatars. This broadness is not due to the review being confined to one specific technological implementation but to the focus on the common psychological question of self-referential appearance representation as opposed to any individual technological implementation: The four sub-types already identified in the eligible corpus are explicitly differentiated in Table 2 and analyzed separately when this evidence allows (Section 3.1.3).

This typology is used in the Results (Section 3.1.3) to examine the relation of harms and benefits in Section 3.1.2 and Section 3.1.4 to idealization in general and whether any harms or benefits in the Results are systematically related to idealization without it.

### 2.4. Study Selection

The records were retrieved from the databases, and the duplicates were identified and removed in a reference management system. Screening was then carried out in two phases and by the same method.

**Figure 1:**
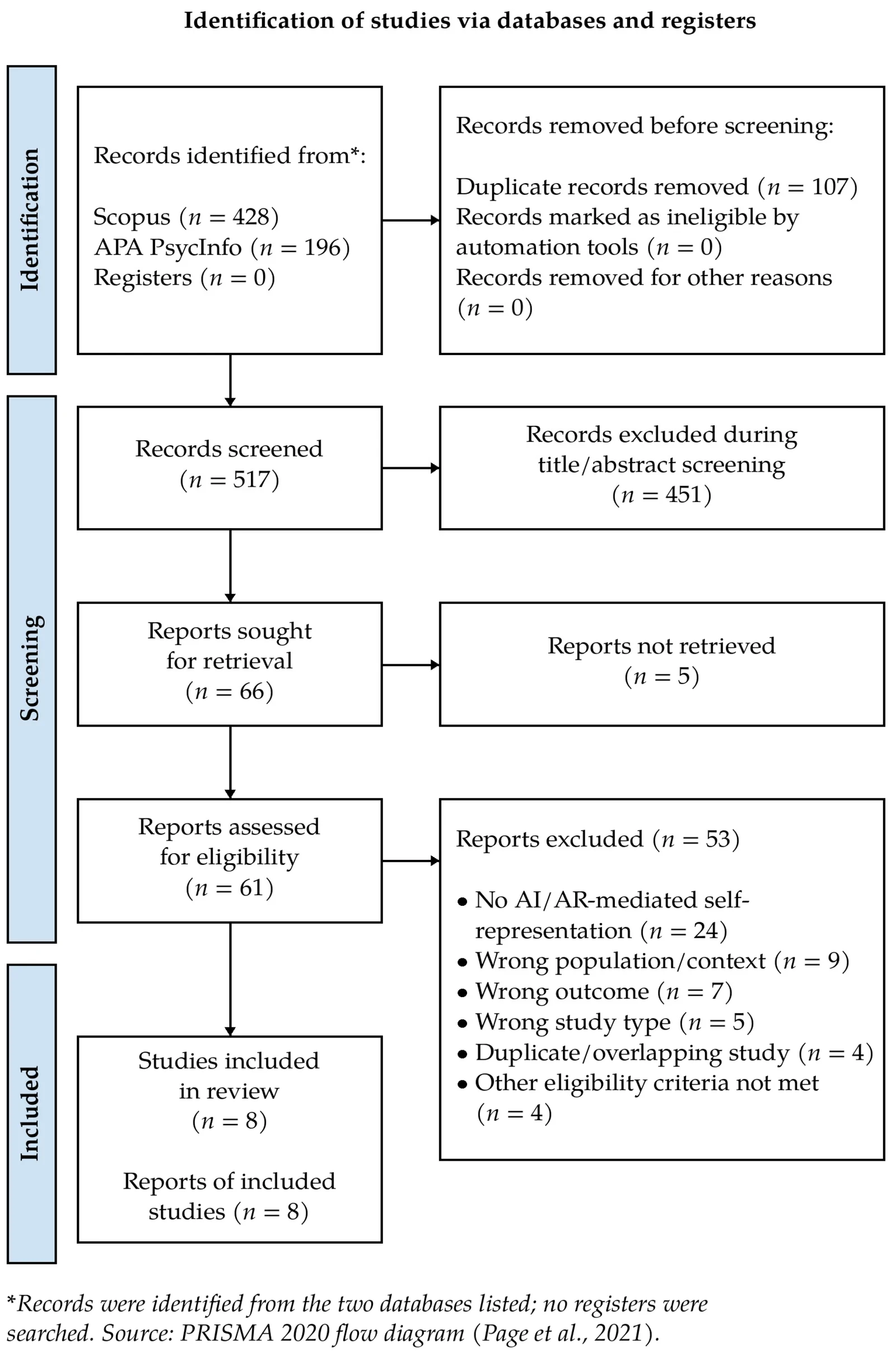
PRISMA 2020 flow diagram of the study selection process. Note. *n* = number of studies. * Records were identified from Scopus and APA. PsycInfo; no registers were searched. Adapted from Page et al. (2021).

Screening, extraction, and quality appraisal were carried out by the first and second authors (A.S. and N.M.). Initially, the first author (A.S.) screened all records by the title/abstract for eligibility according to the criteria listed in Table 1. To ensure that no study was misjudged in excluding it, the second author (N.M.) independently reviewed all those records which, at this stage, were excluded; this did not apply to those records kept for full-text review. At the full-text stage, the first author identified all reports that met the inclusion criteria, and the second author independently examined all reports excluded at this stage for the same purpose. In both the first and second stages, any study the second author considered as potentially misreported was discussed by both authors and re-evaluated against the eligibility criteria and, where necessary, the full text until consensus was reached. No formal third-party adjudication was required. We acknowledge this as a limitation of the review process as there was no record of inter-rater agreement at either stage. There was no automation used throughout. The reasons for exclusion at the full-text stage are summarized in Figure 1 of the PRISMA 2020 flow diagram. A total of eight studies were included in the synthesis that met all eligibility criteria.

### 2.5. Data Extraction

A standardized data-extraction form was designed to help standardize the data across studies. The following information was extracted from each article: the author(s), the year of publication, the country in which the study was carried out, the characteristics of the sample, the study design, the technological context and the type of self-representation, the key variables, the theoretical frameworks invoked by the primary authors, the main findings, and any protective psychological constructs examined.

The extraction form was not formally piloted, but the two studies included in the extraction of data’s field structure were independently reviewed by the first and second authors using two included studies prior to the beginning of the extraction process, and the form was adhered to consistently thereafter.

All studies included were extracted by the first author (A.S.). All extracted data were checked independently by the second author (N.M.) with the full text of each report. Content discrepancies between the extractors were addressed by going back to the source report, and consensus was reached through discussion. Study investigators were not contacted for data, and no automation tools were used in screening, extracting or appraising. Cross-study comparison and thematic synthesis were facilitated by extracting data that were organized in a study characteristics matrix.

### 2.6. Evaluation of Quality

The methodological quality of included studies was assessed using the Mixed Methods Appraisal Tool (MMAT, 2018) (Hong et al., 2018). The MMAT was created specifically for evaluating both qualitative and quantitative, as well as mixed methods research, with a single instrument for reviews conducted with mixed methods.

The studies were assessed using the MMAT criteria for each design type: relevance of the research questions, research sampling, quality of research measures, extent of data, and consistency of data collection and interpretation. The quality assessment was not used to exclude studies from interpretation, but solely for informational purposes.

The first author (A.S.) carried out the quality appraisal using the MMAT. The second author (N.M.) reviewed all criterion-level judgments against the source reports, and discrepancies were resolved by discussion. As recommended by MMAT, no overall numerical quality score was defined, and appraisal was used to interpret findings, not to exclude studies.

### 2.7. The Risk of Bias Assessment Covers the Following Areas:

The MMAT framework was used to evaluate the risk of bias as part of quality appraisal. Special attention was paid to selection bias, sampling limitations, self-report bias, measurement bias, and reporting bias as potential sources of bias. Studies with methodological weaknesses were included because of the diversity of the types of studies in this rapidly expanding literature, but the biases were identified and taken into account when interpreting the results and conclusions.

### 2.8. Synthesis of Data

The variability in the research design, population, technological context and outcome measures among the studies included in the analysis precluded the use of a meta-analysis. Narrative thematic synthesis was therefore carried out.

Coding was in two stages: The first phase involved two independent coders coding each of the studies’ reported variables, mechanisms, and findings, close to the terms used by the main authors and not limited to the three guiding theories; this phase was designed to capture any construct/mechanism that was reported in the primary studies, whether it fell under the three guiding theories or not. Codes generated by both the authors were compared, and any discrepancies in code labeling were discussed and resolved. In the second phase, the codes produced in the first were clustered together in larger patterns and arranged according to the three guiding theoretical frameworks that were used throughout the review to organize and interpret the relationships between codes, but not as a means to decide which codes would be retained. The four themes that emerged from this process included: AI-enhanced selves, digital idealization, self-discrepancy, and the construction of the ideal self, as well as protective and contextual factors.

Although the coding openness of the construct is not of primary interest in the present study, we note that this two-phase approach, irrespective of the level of coding openness of the construct, would have surfaced constructs outside the three guiding theories, but not those that are not present, or are only sparsely represented, across the eight included studies; e.g., positive body image was not measured as an outcome in any of the eight included studies. This will be addressed in more detail in Section 4.2.

## 3. Results

A total of 624 papers were found in the two database searches (Scopus, *n* = 428; APA PsychInfo, *n* = 196). One hundred and seven duplicate records were eliminated, leading to a total of 517 records screened by title and abstract, with 451 being obviously ineligible. Sixty-six reports were sought for retrieval, with five reports not being retrievable, and the full text of the remaining 61 reports was assessed. Fifty-three reports were excluded at this stage, mostly due to other eligibility criteria not being met (*n* = 4), wrong outcome (*n* = 7), wrong study type (*n* = 5), duplicate or overlapping study (*n* = 4), wrong population or context (*n* = 9), and technology not involving self-representation using AI or AR (*n* = 24) (Figure 1). The original single-database search, plus the search for additional APA PsycInfo records, and the search using the updated search terms with positive body image and body appreciation constructs were found to contain no additional eligible studies, leaving eight studies.

Figure 1 Study selection process followed in the present study using the PRISMA 2020 flow diagram. Identification: Scopus *n* = 428, APA PsycInfo *n* = 196, registers *n* = 0, duplicates removed *n* = 107; screening: studies screened *n* = 517, studies excluded based on title/abstract *n* = 451, studies for which reports were sought *n* = 66, studies for which reports were not retrieved *n* = 5, studies assessed for eligibility *n* = 61, studies excluded at full text *n* = 53 (no AI/AR-mediated self-representation *n* = 24, wrong population/context *n* = 9, wrong outcome *n* = 7, wrong study type *n* = 5, duplicate/overlapping study *n* = 4, other eligibility criteria not met *n* = 4); included: studies *n* = 8, reports of included studies *n* = 8.

### 3.1. Overview of Included Studies Included Studies

This synthesis is based on eight empirical studies: one randomized experiment (Schroeder & Behm-Morawitz, 2025), one qualitative study (Kumar & Agarwal, 2025), two cross-sectional quantitative studies (Caravelli et al., 2025; Garg et al., 2026), three mixed methods (Fiedler et al., 2025; Javornik et al., 2022; Sinlapanuntakul & Zachry, 2025), and one multi-study experimental report that combines three experiments and one survey study (Javornik et al., 2021). The methodological quality of the studies, assessed using the MMAT, was generally acceptable for measurement validity and statistical rigor, but for sample representativeness, there were some recurring ‘can’t tell’ and ‘no’ judgments in the studies, and for blinding/randomization-procedure reporting, there were some recurring ‘can’t tell’ and ‘no’ judgments. Therefore, causal and population-level claims below are made with the corresponding caution.

The thematic synthesis yielded four themes: (1) self-representations enhanced by AI/AR as a novel source of the ideal self; (2) the self-discrepancy mechanism as a pathway connecting self-representations to appearance-related distress; (3) the nature of the self-representations as a boundary condition, differentiating idealized from transformed or true from self-similar or neutral self-representations; and (4) protective and contextual factors as buffers or reversals of the self-discrepancy pathway.

#### 3.1.1. The Ideal Self Is a Novel Source of Self-Representation Through AI/AR

AI- and AR-mediated self-representations, without exception, served as a means of creating an idealized version of self as opposed to comparison to others throughout the whole corpus. The use of AI beauty filters was directly predicted by ideal self-congruence (β = 0.538, *p* < 0.001) and ideal attractiveness (β = 0.290, *p* < 0.001), showing that the use of AI beauty filters is driven by the desire to appear a certain way, rather than simply to look pretty (Garg et al., 2026). Javornik et al. (2022) made this distinction explicit at the level of the concept of self-representation itself and distinguished between three different AR filter use categories: true, ideal, and transformed self-representation, the latter group being different from the former two on all the downstream outcomes detailed in 3.1.3. In line with Kumar and Agarwal’s (2025) grounded-theory analysis, AR filters also had an active effect of “redefining/enhancing the ideal standard of attractiveness” for the users, as they made the gap between actual and ideal larger by comparing with others’ filtered images.

#### 3.1.2. The Self-Discrepancy as Central Mechanism Linking AI-Enhanced Representations to Distress

Self-discrepancy has been the most consistently supported link between the appearance aspects of AI/AR representations and negative outcomes, evidenced across three independent designs. In the only randomized experiment in the corpus, Schroeder and Behm-Morawitz (2025) found that self-presentation through a slimming AR filter caused significantly more state social comparison than a presentation of another person through a filter, and that these effects of the filter on body-ideal discrepancy, desire for weight loss, self-objectification, and anti-fat attitudes were mediated by body dysmorphic cognitions. Garg et al. (2026) conducted two survey studies and applied structural equation modeling to the data, revealing that the use of AI beauty filters significantly predicted lower body-image state (β = 0.650, *p* < 0.001), higher fear of negative appearance evaluation (β = 0.388, *p* < 0.001), and lower appearance self-esteem (β = 0.252, *p* < 0.001) (Garg et al., 2026). In three consecutive experiments, Javornik et al. (2021) demonstrated that AR mirror and virtual makeup try-on technology created a larger gap between ideal and actual attractiveness, and that the direction and magnitude of the gap depended on the appearance self-esteem of the participants at the start of each experiment (Javornik et al., 2021). To this convergent cross-sectional evidence, Caravelli et al. (2025) added that in the present study, 18% met the clinical cutoff for probable body dysmorphic disorder/eating disorder symptomatology, with appearance-improving filter use also being significantly correlated with body-image concern and dissatisfaction with one’s face.

#### 3.1.3. Representation Type as Boundary Condition

Building on the techno typology outlined in Table 2, this section explores whether self-discrepancy and its downstream consequences are related to idealization alone or are moderated by technological sub-type beyond idealization. One of the salient features that make this synthesis different from previous appearance-technology studies is that the self-discrepancy and its downstream consequences were only salient in idealized self-representations and not in all other self-representations. Among 536 active AR filter users, Javornik et al. (2022) found that idealized self-presentation was associated with reduced self-acceptance (B = −0.269, *p* < 0.001), while true self-presentation and transformed (non-idealizing) self-presentation was associated with increased self-acceptance (B = 0.145, *p* = 0.009 and B = 0.148, *p* = 0.004, respectively) and positive affect (B = 0.103, *p* = 0.038 and B = 0.160, *p* = 0.002, respectively). Since the study participants were not put into a filter/no-filter condition but were self-selected on a continuous motivational scale, the findings of that study suggest that the motivational valence of filter use depends on the motive, not that idealized use is harmful compared to non-use. The former distinction was strengthened by Fiedler et al. (2025), who found that exposure to a self-similar (but not idealized or slimmed) virtual human also significantly raised sense of embodiment and self-identification compared to a generic virtual human, which modified the body-weight perception without the degradation in appearance that has been reported in idealized-filter studies (Fiedler et al., 2025). In fact, in the same model, appearance-enhancing filters were significantly associated with body dissatisfaction, whereas “goofy”, entertainment-focused filters were not (Caravelli et al., 2025).

#### 3.1.4. Protective and Contextual Factors

Within the corpus, the protective and buffering effects for the protective constructs were not uniformly distributed, and this imbalance needs to be acknowledged in claims made about the individual constructs within each category.

Body-image-specific constructs: This was the same following the updated search (which included the use of positive body image terminology) and the inclusion of APA PsycInfo as a second database (Section 2.2), where no studies directly measured positive body image or body appreciation as defined by Tylka and Wood-Barcalow (2015b). The current evidence base does not provide an answer to this, but it is considered in more detail in Section 4 and Section 4.2.

Domain-general self-related resources: Two studies investigated appearance self-esteem directly. Garg et al. (2026) discovered that the usage of AI beauty filters was a significant predictor of lower appearance self-esteem (β = 0.252, *p* < 0.001) (Garg et al., 2026). Javornik et al. (2021) showed that the direction and nature of the ideal–actual gap was influenced by participants’ appearance self-esteem scores prior to the intervention, and that AR use could indirectly boost self-compassion by first closing the ideal–actual gap, but that this effect was contingent on baseline self-esteem scores (Javornik et al., 2021). Thus, in just one study, self-compassion was only shown to be a downstream, conditional buffer, rather than being tested as an independent buffer. Across the eight included studies, resilience and emotion regulation were not measured as protective constructs and are not presented as review findings because they are based on the theoretical background presented in this study and remain a hypothesis for future empirical testing in the context of this study.

Regulatory processes: There is no direct measure of cognitive reappraisal, or other adaptive emotion-regulation strategies, as protective mechanisms in studies included in the corpus.

Contextual and technological features: The type with the best evidence in the corpus is this one. In collaborative goal-directed tasks, as compared to traditional video, avatar-mediated representation significantly improved self-esteem (d = 1.05) and collaboration satisfaction (d = 0.74) (Sinlapanuntakul & Zachry, 2025). Javornik et al. (2022) reported that positive affect and self-acceptance were related to true/transformed (non-idealizing) self-presentation, whereas self-presentation idealization was related to decreased self-acceptance. Fiedler et al. (2025) showed that a non-slimmed but also self-similar virtual human led to greater embodiment and self-identification without involving the degradation of the appearance, as it would with idealizing filters (Fiedler et al., 2025). Some of the participants used the filters for self-enhancement of creativity, not for concealing appearance, and were inspired by them, not making them feel bad due to the process of comparison (Kumar & Agarwal, 2025). In all four studies, the protective pattern was consistently found: representation type, use motivation, and task framing were the factors that either broadened or narrowed the actual–ideal gap, but not a general psychological trait.

A taxonomic classification of the constructs, evidentiary status of the constructs, and the supporting study of the constructs that were identified in the corpus are summarized in Table 3. The characteristics of the studies used are presented in Table 4.

### 3.2. Methodological Quality of Included Studies

The methodological quality of the studies included was evaluated using the Mixed Methods Appraisal Tool (MMAT) version 2018. The appraisal criteria were chosen based on the methodological design of each study, with randomized experimental studies being judged according to the criteria of the randomized controlled trial; qualitative studies according to the qualitative criteria; quantitative descriptive studies according to the quantitative descriptive criteria; and mixed-methods studies on their qualitative, quantitative and mixed-methods parts. Appraisal was done at the criterion level with no numerical quality score being calculated. The outcome of the methodological quality assessment is presented in Table 5, and detailed MMAT criterion assessments of the included studies are presented in Table 6, detailed criterion-specific assessments are presented in Table 7, Table 8, Table 9 and Table 10.

Overall, the included studies demonstrated generally acceptable methodological quality across their respective designs, although several limitations were identified. The qualitative dimension of Fiedler et al. (2025) was given the “Can’t tell” ratings in terms of the adequacy of derivation and substantiation of findings, mostly due to the lack of a full thematic analysis and the lack of verbatim quotations (Fiedler et al., 2025). The most common quantitative limitations were those related to representativeness and possible nonresponse bias, especially in cross-sectional survey designs. Garg et al. (2026) fulfilled the sampling-relevance and analytic-rigor criteria, but, as the authors themselves noted in their own limitations, representativeness could not be ensured as a purposive sampling of a single-platform user base (mostly Indians) was used (Garg et al., 2026). The randomized experimental studies showed full reporting of outcomes and compliance with study procedures, but blinding was not possible, and the methods for randomization were not fully described. The integration was well represented in Sinlapanuntakul and Zachry (2025) and Javornik et al. (2022), while in Fiedler et al. (2025), integration was less clearly represented (Fiedler et al., 2025; Javornik et al., 2022; Sinlapanuntakul & Zachry, 2025).

## 4. Discussion

This review explored whether AI-enhanced self-representations could be a unique type of ideal self, investigated the nature of the psychological pathway through which AI-enhanced self-representations are involved, and examined the relationship between positive body image and other constructs linked to the relationship between self-representations and the ideal self. In the eight studies, which were gathered by searching two databases and that corresponded to four distinct sub-types of self-representations as a function of the technology used (see Table 2), the evidence does not support the uniform picture that has been advanced by the previous appearance-technology-based literature, but rather a qualified, boundary-conditioned picture.

*AI/AR representations as distinctive ideal selves.* Consistent with the corpus, AI- and AR-mediated self-representations are not just another type of idealized other with which people are expected to compare themselves as predicted by Social Comparison Theory (Festinger, 1954). Rather, in most of these studies, the comparison target was typically the individual’s own image (improved, filtered, or changed). This self-referential ideal is actively being created through the use of AI/AR technologies, as shown by both Garg et al. (2026) and Kumar and Agarwal (2025). Schroeder and Behm-Morawitz (2025) offer the corpus’s strongest causal evidence that comparing oneself to one’s own filtered image produces a distinct and more potent comparison process than comparing to another person’s filtered image. These results tentatively indicate that self discrepancy theory may be expanded to address the technologically mediated ideal self, which is not accessed through observing other people’s media images, but through personal manipulation of one’s self-image. More rigorous empirical research is needed to determine whether an additional extension of self discrepancy theory is warranted.

*Representation type, not technology exposure, emerges as a potential boundary condition.* Applying the technological typology introduced in Table 1, the clearest pattern this review identifies, relative to prior syntheses, is that harm associated with AI/AR self-representation appears concentrated in idealized representations specifically, not on the category of AI/AR-mediated self-representation as a whole. When Javornik et al. (2022) surveyed 536 active AR users, they discovered that the strength of the ideal self-presentation motive was significantly associated with a decrease in self-acceptance (β = −0.269, *p* < 0.001), while both the true self-presentation motive and the transformed self-presentation motive predicted significantly higher levels of self-acceptance and positive affect. This result does not imply that idealized use is harmful as compared to no use, as all the subjects in this study were already filter users and thus were rated on motivational continuums rather than given filter versus no-filter conditions. Two other related types of technology—the self-similar virtual human and user-configured avatars—are extended in similar ways by Fiedler et al. (2025) and Sinlapanuntakul and Zachry (2025), respectively, with realism, collaboration, and user-control types of representation being linked with neutral-to-positive results. Combined, these findings suggest the alternative hypothesis for the theoretical question raised by this review: AI-mediated self-representation can lead to a greater self-discrepancy, which is not necessarily the case when self-representation involves non-idealizing AI. This is provided here as a testable extension of self-discrepancy theory for future confirmatory studies, but not as an effect already confirmed by the current synthesis.

*Protective and contextual factors operate unevenly across construct categories, not as a single buffering mechanism.* In this corpus, protective evidence could be seen in the fourth category (contextual/technological characteristics; Table 3), with the majority of instances occurring in this domain. Sinlapanuntakul and Zachry (2025), Javornik et al. (2022), Fiedler et al. (2025), and Kumar and Agarwal (2025) all suggest that it was not the same general psychological trait that caused the actual–ideal gap to open or close, but rather the representation type, use motivation, and task framing. Appearance self-esteem was found to be a genuine modulator in two studies (Garg et al., 2026; Javornik et al., 2021), while self-compassion was a conditional, downstream outcome in one study (Javornik et al., 2021). While resilience and emotion regulation were mentioned in the theoretical background of this review as possible protective resources, none of the eight studies included measured and reported them as protective constructs and thus cannot be interpreted as review findings. The qualitative and mixed-methods evidence for these protective and boundary-condition themes, including Fiedler et al.’s (2025) self-similar virtual human findings and Sinlapanuntakul and Zachry’s (2025) avatar findings, alongside Javornik et al. (2022) and Kumar and Agarwal (2025), meet MMAT criteria more consistently than the cross-sectional quantitative evidence discussed.

*Positive body image: an identified gap at the intersection of two bodies of literature.* Positive body image or body appreciation (Tylka & Wood-Barcalow, 2015b) was not measured in any of the studies included in the present study. This was not assumed but directly tested: after implementing the supplemental search in Scopus and APA PsycInfo (Section 2.2) with positive body image and body appreciation language, 624 records were identified; 517 remained after deduplication, and 61 were assessed at full text, but none were found eligible. Interestingly, the majority of the remaining records excluded for the reasons listed above were not completely without any representation of the body as AI/AR, but because there was no self-representation component of the body (*n* = 24 of 53 exclusions). This implies that the gap that emerged from this review is in the context of self-representation, mediated by AI and positive body image research, and not inadequate search coverage of the literature alone or search coverage of body image research alone. A study immediately after the end of the review’s search window assessed body appreciation using the Body Appreciation Scale-2 (Tylka & Wood-Barcalow, 2015a) and experimental exposure to AR beauty filters on one’s own and others’ facial images, a focus similar to the present review (the same focus as body appreciation was measured, but not as an outcome of the filter exposure). This indicates that the field is starting to move towards correlating positive body image measures in just this experimental setting, confirming the timeliness of the gap noted here as well as the importance of testing this relationship directly, as a first outcome, in future research. The overlap between constructs like protective filtering and body appreciation, and the algorithmic idealization of appearance as a critical feature of this technology, is an obvious and specific direction for future empirical research, which is discussed further below.

*Calibrating conclusions to evidence quality***.** The strongest causal claim in this synthesis, that self-comparison to one’s own idealized image elicits more distress than does self-comparison to another’s idealized image, is based on one randomized experiment (MMAT appraisal, Table 3), which, at best, can be considered an initial causal finding rather than a confirmatory finding (C1: CT; C2: CT) due to the lack of blinding and randomization procedures. In the cross-sectional (quantitative) studies that have been used to support the self-discrepancy pathway (Caravelli et al., 2025; Garg et al., 2026), particularly when they use purposive and single-platform samples and a geographically concentrated sample, these studies should be interpreted as revealing association within the sample and not as being representative of population-level effects. The qualitative and mixed-methods evidence supporting the protective and the boundary-condition themes is more consistent with the MMAT and comparatively stronger in supporting the above distinction between representation-type.

### 4.1. Implications

Theoretically, the review indicates that SDT might be expanded to differentiate between idealizing and non-idealizing technologically mediated self-representations, and that positive body image constructs such as protective filtering and body appreciation are a theoretically motivated but untested addition to models of AT use. Idealization is the result of this synthesis as a possible boundary condition between appearance technologies and psychological harm that needs to be directly tested empirically in future confirmatory studies, but not as a proven mechanism. However, future confirmatory studies should directly test this distinction either by varying the idealization level (with technology type held constant), as was done with the motivational scale approach by Javornik et al. (2022), or by including validated measures of positive body image (e.g., the Body Appreciation Scale-2; Tylka & Wood-Barcalow, 2015a), which would allow for testing the moderation of the pathway from idealization to distress found herein. In practice, instead of general bans on AI/AR self-representation technologies, the results suggest a set of appearance-technology design decisions, such as the automatic use of presets instead of idealizing images, could be a more effective point of intervention.

### 4.2. Limitations

There are some limitations in this review: some inherent in the developing literature and others related to the process of the review. The number of studies in the final corpus is small (k = 8), and there was only one randomized experiment, with cross-sectional quantitative evidence being the prevalent data type, thus reducing causal inferences. The included samples were concentrated geographically (India; U.S.; U.K.) and were recruited through purposive or convenience sampling, which reduces generalizability, an issue that the primary studies themselves also acknowledge. The four themes synthesized here were grounded in a two-phase coding process that took place independently of the review’s three guiding theories during its first phase (Section 2.8); however, this reflects a narrative rather than a meta-analytic synthesis, since the eight studies were heterogeneous in design, technology, and outcome measures, and the coding process cannot surface a construct—such as positive body image—that is simply not included in the outcome measures of the available literature, irrespective of how open the coding was.

The first author screened all records, reports, and extracted data, and conducted quality appraisal for the eight included studies. The second author independently verified exclusions at both the title/abstract and full-text screening stages—that is, records or reports excluded by the first author, rather than the full set—and separately reviewed all quality-appraisal judgments against the source reports; no inter-rater agreement statistic was recorded at the screening stage. This model affords some protection against the wrongful exclusion of eligible studies, but it is not as robust as full duplicate independent screening, extraction, and appraisal by a larger review team, and this should be considered when interpreting the reproducibility of the review’s conclusions.

Lastly, although the search was expanded to two databases and revised to include positive body image terminology, it remains limited to what these two databases index—meaning relevant evidence may exist in databases not searched, in non-English-language publications, or in disciplines outside the psychology, communication, and human–computer interaction literature that Scopus and APA PsycInfo cover. Beyond this question of completeness, the choice of these two databases may also have shaped which studies were found, not only how many. Both databases favor English-language, journal- and proceedings-based literature within psychology, communication, and adjacent social-science fields, which may partly explain why the eight included studies are concentrated in India, the U.S., and the U.K., and why no study measuring positive body image or body appreciation was identified. Because the four themes in this review come from this particular set of eight studies, a differently composed corpus—for instance, one drawing on stronger coverage of non-English or non-Western body-image research—could plausibly have emphasized the themes differently or surfaced constructs the current search did not capture. This composition effect is worth weighing alongside completeness when judging the thematic conclusions of this review, not treated merely as a reason more studies might exist elsewhere. Since the search cutoff in May 2026, at least one relevant study using a validated positive body image measure in a closely related experimental context has been published (Dietel & Vocks, 2026), underlining how quickly this literature is moving even as this review goes to press.

## 5. Conclusions

The review aimed to identify the effects of self-representations mediated by AI and AR technologies by synthesizing eight empirical studies that were identified through a two-database search, where negative and positive body-image constructs were included, to examine whether AI- and AR-enhanced self-representations are a unique type of the ideal self. The results indicate that they do, but only when the AI/AR representations are idealizing, not when they are true, transformed, self-similar, or collaboratively framed; self-discrepancy and appearance-related distress emerged when the representations were idealizing, whereas a true, transformed, self-similar, or collaboratively framed representation was linked to neutral or protective results, such as greater self-acceptance, embodiment, and self-esteem. In a subset of studies, appearance self-esteem and, tentatively, self-compassion appeared to moderate this pathway; resilience and emotion regulation were not demonstrated as protective factors in the included body of research. However, positive body image and body appreciation were not assessed in any of the studies included, and an expanded search did not reveal this to be due to a lack of search coverage of the literature, but rather to the fact that it was a genuine gap at the intersection of two well-defined bodies of literature. Because there is little current evidence available and it has a fairly large degree of methodological variation, these statements should be viewed as the initial result of a theoretically grounded model, limited to the boundaries of the available evidence, rather than a proven causal model. Randomized designs directly manipulating idealization level, employing positive body image measures with more demographically and geographically diverse samples are desired to help test this account of AI-enhanced ideal selves.

## Figures and Tables

**Table 1:** Inclusion and exclusion criteria.

Criteria	Inclusion	Exclusion
Publication Type	Peer-reviewed journal articles and full papers published in archival, peer reviewed conference proceedings	Conference abstracts and extended abstracts, editorials, commentaries, book chapters, opinion pieces, dissertations, theses
Publication Year	January 2021–May 2026	Published before January 2021
Language	English	Publications not available in English
Information Source	Records retrieved from the databases searched (See Section 2.2)	Records not retrievable in full text
Study design	Empirical quantitative, qualitative and mixed methods empirical studies	Conceptual and theoretical papers, literature reviews, systematic reviews, and meta-analysis
Population	Adolescents, young adults, and adults	Studies not involving humans
Technology context	Technologies that generate or modify a visual representation of user’s own appearance, including AI beauty filters, AR face and body filters, computationally generated self- representations, self-similar virtual humans and user- configurated avatar (See Section 1 and Table 2 for operational definition and typology)	Technologies that do not represent the user’s own appearance (e.g., exposure to images of other people, generic non-self-virtual characters, text-based AI); appearance modification without a digital representational component
Outcomes	self-discrepancy or actual–ideal self-gap, body image (negative and positive), body appreciation, appearance anxiety or fear of negative appearance evaluation, appearance related social comparison, self-objectification or body surveillance, self-acceptance, protective psychological constructs such as self-compassion, self-esteem, resilience, emotion regulation	Studies reporting no psychological outcome related to body image, appearance evaluation, or self-concept.

**Table 2:** Technological sub-types of AI-enhanced self-representation included in this review.

Sub-Type	Underlying Process	Modality	Idealization by Default	Studies
AI beauty filter	ML-based facial landmark detection; generative modification of a static image	2D, photo-based	Typically idealizing (skin smoothing, symmetry, slimming)	Garg et al. (2026); Kumar and Agarwal (2025)
AR real-time filter	Computer-vision tracking; real-time transformation of live video	2D/2.5D, live	Varies by filter design (idealizing to neutral/entertainment)	Schroeder and Behm-Morawitz (2025); Caravelli et al. (2025); Javornik et al. (2021, 2022)
Self-similar virtual human	3D body-scan-based rendering	3D, rendered	Non-idealizing in the tested condition (self-similar, not slimmed)	Fiedler et al. (2025)
User-configured avatar	User-selected stylized features tracking live movement/expression	2D animated overlay	Non-idealizing (stylized, user-controlled)	Sinlapanuntakul and Zachry (2025)

**Table 3:** Protective and contextual constructs, by taxonomic category and evidentiary status.

Category	Construct	Evidentiary Status in This Corpus	Supporting Studies
Body-image-specific	Positive body image/body appreciation	Not measured in any included study	—
Domain-general	Appearance self-esteem	Measured directly; moderates discrepancy pathway	Garg et al. (2026); Javornik et al. (2021)
Domain-general	Self-compassion	Measured as a conditional downstream outcome in one study only	Javornik et al. (2021)
Domain-general	Resilience	Not measured; discussed only in the background literature	—
Regulatory	Emotion regulation/cognitive reappraisal	Not measured in any included study	—
Contextual/technological	Non-idealizing or self-similar representation	Directly evidenced; associated with self-acceptance, embodiment	Javornik et al. (2022); Fiedler et al. (2025)
Contextual/technological	Collaborative/goal-directed use context	Directly evidenced; increased self-esteem and satisfaction	Sinlapanuntakul and Zachry (2025)
Contextual/technological	Creative self-enhancement motivation	Reported qualitatively as a protective pattern	Kumar and Agarwal (2025)

**Table 4:** Characteristics of the included studies.

Study	Year	Sample	Technology	Own Self Representation	Design	Variables	Main Findings	Theme
Schroeder & Behm-Morawitz	2025 (online 2024)	N = 187 US social media users (adults 19–66, M = 36.28); majority white (*n* = 153) and women (*n* = 156); recruited via ResearchMatch	AR slimming beauty filter (Snow app) vs. neutral color filter	Yes—Condition 1 used filter on own image (self-representation); Condition 2 watched another person use it (other-representation); Condition 3 used a neutral color filter on own image (control)	Online experiment; 3-condition between-subjects random assignment (*n* = 63 own-filter, *n* = 79 watch-other, *n* = 45 color-filter control)	IV: condition (slim filter-self/watch-other/color filter). Mediators: state comparison (social self-comparison for own-image conditions; social comparison for watch-other condition), body dysmorphic cognitions. DVs: body ideal discrepancy, desire for weight loss, self-objectification, anti-fat attitudes, preference for filtered image. Covariate: social media use	Using a slimming filter on one’s own image produced the strongest state comparison (social self-comparison) greater than watching someone else use it. State comparison mediated effects of condition on self-objectification, desire for weight loss, and anti-fat attitudes (but not body ideal discrepancy or filtered-image preference). Body dysmorphic cognitions mediated condition’s effect on all outcome variables (body ideal discrepancy, desire for weight loss, self-objectification, anti-fat attitudes, preference for filtered image), primarily when comparing slimming filter to color-filter control. Slimming filter use also directly predicted greater preference for the filtered image over one’s real appearance.	Introduces social self-comparison as a novel extension of social comparison theory—comparing one’s real self to an AI/AR-filtered version of the self is a distinct and more potent comparison process than comparing to others; body dysmorphic cognition acts as a key mechanism linking filter/AI self-representation exposure to negative body-image and appearance-related outcomes
Fiedler et al.	2025	N = 60 participants. Participants were exposed to photorealistic virtual humans generated using their own body scans. The study also examined participants’ BMI, self-esteem, body shape concerns, and gender as individual factors.	Augmented reality (AR) using photorealistic virtual humans. Virtual humans were either self-similar to the participant or generic. In controlled conditions, the virtual human’s movements were synchronized with the participant’s movements; in non-controlled conditions, it moved independently. Body weight could also be modified by ±35% of the participant’s actual body weight.	Yes—strongly. The self-similar virtual human was generated from a scan of the participant’s own body, creating a personalized virtual representation. The study specifically examined self-similarity, self-identification, and sense of embodiment.	Experimental 2 × 2 mixed-design study. Factors were self-similarity (self-similar vs. generic) × motor control (controlled vs. non-controlled). Participants completed body-movement and body-weight estimation tasks, questionnaires assessing embodiment/self-identification, and semi-structured interviews.	Sense of embodiment (SoE); self-identification; self-attribution; perceived self-similarity; body-weight perception/estimation; BMI; self-esteem; body-shape concerns; gender.	Self-similarity significantly increased sense of embodiment and self-identification. Participants estimated the body weight of self-similar virtual humans more accurately than generic virtual humans. Motor control had weaker effects and did not significantly change body-weight perception. Participants’ BMI, self-esteem, and body-shape concerns also influenced body-weight estimation. Self-similar virtual humans produced significantly greater perceived self-similarity and self-attribution than generic virtual humans.	Personalized virtual self/self-identification and embodiment; virtual body perception; body-image-related individual differences. More specifically: self-similar digital self → stronger embodiment/self-identification → altered body perception.
Garg et al.	2026	Two complementary cross-sectional studies. Study 1: N = 468 AI beauty-filter users in India: 175 male (37%) and 293 female (63%), with participants across different age groups. Study 2: N = 312 users of BeautyPlus Cam–AI Photo Editor; total N = 780	AI beauty filters integrated into social-media platforms (Study 1) and BeautyPlus Cam–AI Photo Editor specifically (Study 2). Filters digitally modify/enhance facial appearance, including skin smoothing, facial contours and virtual makeup.	Yes. Users apply AI filters directly to their own faces/photos, creating an enhanced/idealized version of themselves. The study explicitly conceptualizes the filtered image as creating a discrepancy between the actual self and ideal self.	Two cross-sectional quantitative survey studies. Study 1 used purposive sampling of AI beauty-filter users across Instagram, Facebook and Twitter, with SEM used to test the hypothesized relationships. Study 2 replicated/extended the model in the specific BeautyPlus app context.	Ideal self-congruence (ISC); ideal attractiveness (IA); AI beauty-filter use (AIU); lower body-image state (BIS); fear of negative appearance evaluation (NAE); low appearance self-esteem (LAS); body surveillance (BS); gender. The study also examines mediation through BIS and NAE and moderation by BS and gender.	Ideal self-congruence (β = 0.538) and ideal attractiveness (β = 0.290) significantly predicted AI beauty-filter use. AI beauty-filter use significantly predicted lower body-image state (β = 0.650), fear of negative appearance evaluation (β = 0.388), and low appearance self-esteem (β = 0.252), all *p* < 0.001. Lower body image predicted fear of negative appearance	AI-enhanced ideal self → self-discrepancy → appearance-related distress. Key subthemes: ideal-self motivation; negative body-image state; appearance anxiety/fear of negative evaluation; low appearance self-esteem; body surveillance as a vulnerability/moderating factor.
Kumar & Agarwal	2025	N = 18 AR-filter users from India, recruited from Delhi/NCR. Participants were Millennials and Gen Z; mean age = 23 years, with all but four aged ≤24. Participants had used Snapchat/Instagram for >1 year and AR filters extensively during the previous year.	AR-based social-media filters, particularly filters used on Snapchat and Instagram to alter users’ appearance in selfies/photos/videos.	Yes. Filters modify participants’ own appearance, including skin tone, hair color, makeup and facial/body appearance. The study specifically examines how this altered digital representation affects the actual and ideal self-concept.	Inductive qualitative study using Charmaz’s grounded theory approach. Data were collected through 18 semi-structured interviews, conducted by telephone/Google Meet between 3 June and 17 July 2021. Theoretical saturation was reached after the 14th interview, followed by four additional interviews.	Actual self-concept; ideal self-concept; ideal–actual self-gap; social comparison; body satisfaction/body dissatisfaction; self-confidence; envy; inspiration; self-enhancement; well-being; usage intention.	AR filters broadened the ideal–actual self-gap in two ways: they redefined/enhanced the ideal standard of attractiveness and, through social comparison, could diminish perceptions of the actual self. Participants reported increased social comparison, anxiety, envy and body dissatisfaction. However, some participants experienced inspiration and increased self-confidence/body satisfaction because filters allowed them to creatively enhance their appearance and attempt to narrow the gap.	AR-enhanced ideal self → social comparison → broadened actual–ideal self-discrepancy → appearance/body dissatisfaction. Secondary theme: creative self-enhancement as a potentially positive response to self-discrepancy.
Caravelli et al.	2025	N = 397 recruited; final analytic sample N = 340 undergraduates at San Diego State University (5 non-binary participants excluded due to small cell size); 82.6% cisgender women, 65.2% White, 73.6% heterosexual; Mage = 20.3 (SD = 2.68); all had used TikTok in past 2 weeks	TikTok facial filters—appearance-improving filters and “goofy” (entertainment-oriented) filters, measured via author-developed TikTok Filter Use Scale (TFUS)	Yes—own facial image altered via TikTok face-altering filters	Cross-sectional survey; hierarchical linear regressions (with gender as covariate) testing each filter type independently and jointly; EFA conducted on both new scales (TFUS, Facial Dissatisfaction Scale)	IV: appearance-improving filter use, Goofy filter use; DV: body image concern (BICI), facial dissatisfaction (FDS: face-related and skin/hair subscales); Covariate: gender	Both appearance-improving and goofy filter use were significantly positively associated with body image concern and facial dissatisfaction when tested independently; when entered together, only appearance-improving filter use remained significant, while goofy filter use became non-significant; women reported significantly greater body image concern and facial dissatisfaction than men; 18% of sample met clinical cutoff for probable BDD/ED	Self-discrepancy and appearance anxiety from AI/AR-filtered self-image; motivation for filter use (appearance-enhancing vs. entertainment) as a differentiating risk factor
Sinlapanuntakul & Zachry	2025	N = 32 (8 pre-formed groups of 4); university students/recent graduates with pre-existing relationships (friends/colleagues); 29/32 of Asian heritage; recruited from a US university	Memoji (2D animated, humanoid avatar overlay tracking head/facial movement) via FaceTime, compared against traditional live video (Zoom), used during collaborative game-based tasks	Partial—avatar is a self-designed digital representation, not a photorealistic image; participants categorized their avatar as depicting actual self (*n* = 21), ideal self (*n* = 8), or creative/expressive self (*n* = 3)	Mixed-methods, within-subject experimental design (avatar vs. traditional video, counterbalanced order) with goal-directed collaborative tasks, followed by post-task Rosenberg Self-Esteem Scale and Collaboration Satisfaction measures, plus group interviews (thematic analysis)	IV: representation condition (avatar vs. traditional video); DV: self-esteem (Rosenberg Self-Esteem Scale), collaboration satisfaction (process and decision satisfaction subscales); qualitative: benefits/challenges/adoption themes	Avatar representation significantly increased self-esteem (d = 1.05) and overall collaboration satisfaction (d = 0.74) relative to traditional video; self-esteem correlated strongly with collaboration satisfaction only in the avatar condition; qualitative themes showed avatars reduced self-consciousness and social tension and increased confidence and engagement (consistent with self-discrepancy theory—avatars narrow the gap to one’s ideal self—and the Proteus effect), but introduced challenges in expressing/interpreting emotion and an unexpected “self-prioritization” effect	Protective use of digital self-representation: avatar-mediated reduction in self-discrepancy and appearance-related social anxiety (contrasts with beauty-filter studies showing harm—here altered self-representation functions as a buffer rather than a risk factor)
Javornik et al.	2022	Qualitative: N = 10 UK regular Instagram/Snapchat AR-filter users; 9 female, aged 20–29. Quantitative: N = 536 UK residents aged >18 who had used an Instagram AR face filter within the previous month.	AR face filters on Instagram/Snapchat, including beautifying, appearance-enhancing, transformative, funny and other face filters.	Yes. The study explicitly distinguishes true self, ideal self, and transformed self-presentation. Ideal self refers to an aspirational version of oneself; transformed self represents a possible/virtually modified version of oneself.	Sequential mixed methods: interviews followed by a cross-sectional survey. Interviews used thematic analysis; survey used factor analysis and path analysis/bootstrapping to test relationships between motivations, filter use and well-being.	Self-presentation: true, ideal and transformed self; AR-filter use/exploration; enjoyment; social interaction; convenience; affiliation; silliness; creative content curation; self-acceptance; positive affect/mood.	Ideal self-presentation significantly increased filter-use frequency and significantly decreased self-acceptance (β = −0.269, *p* < 0.001). Its association with reduced positive affect was marginal (β = −0.098, *p* = 0.066). True self-presentation increased self-acceptance (β = 0.145, *p* = 0.009) and positive affect (β = 0.103, *p* = 0.038), although it did not predict frequency/exploration. Transformed self-presentation increased filter exploration (β = 0.148, *p* = 0.004), self-acceptance (β = 0.148, *p* = 0.004), and positive affect (β = 0.160, *p* = 0.002). Thus, AR-filtered self-representation was not uniformly harmful: idealized appearance representation was associated with poorer self-acceptance, whereas true and transformed representations were associated with more positive outcomes.	AI/AR-enhanced self-presentation and self-discrepancy; ideal-self vs actual-self; appearance enhancement; self-acceptance and well-being. Particularly relevant to the ideal-self pathway, where digitally enhanced appearance may make the actual–ideal gap more salient.
Javornik et al.	2021	Multiple experimental studies. Study 3: N = 123 female participants, M age = 24.35 years (SD = 6.66), recruited from a British university.	AR mirror/AR virtual makeup try-on, including pre-designed and customizable virtual makeup applied to the participant’s own face.	Yes. The technology directly modifies the participant’s own facial appearance and examines the relationship between the actual self, ideal self, ideal–actual gap, and ideal-self congruence.	Multi-study experimental research. Studies 1–3 experimentally compared AR mirror/AR try-on conditions with regular mirror/control conditions; Study 3 additionally manipulated customization.	Ideal–actual attractiveness gap; actual attractiveness; ideal attractiveness; ideal-self congruence; appearance self-esteem; self-awareness; variety-seeking; self-compassion; AR use/customization.	AR augmentation affected the ideal–actual self gap, but the direction/mechanism depended on appearance self-esteem and the experimental context. In Study 1, people with high appearance self-esteem showed a significantly larger ideal–actual gap in the AR condition, primarily because they rated their actual attractiveness lower. In Study 2, the gap reduction occurred among lower-self-esteem participants and was attributed to a reduction in the ideal self. Study 3 found that AR customization could influence self-related variety-seeking, with effects again depending on self-esteem. Importantly, the authors report that AR use may indirectly increase self-compassion through reducing the ideal–actual gap, although this pathway was dependent on self-esteem.	AI/AR-enhanced self and self-discrepancy; ideal–actual self gap; appearance self-esteem as a moderator; self-compassion as a protective psychological factor; augmented-self plasticity.

**Table 5:** Overall MMAT appraisal of included studies.

Study	Design	MMAT Category	Quality Appraisal
Schroeder and Behm-Morawitz (2025)	Randomized experiment	Category 2	C1: CT; C2: CT; C3: Y; C4: N; C5: Y
Fiedler et al. (2025)	Mixed methods	Category 5	Qual: Y/Y/CT/CT/Y; Quant: CT/Y/Y/Y/Y; Integration: Y/CT/CT/CT/CT
Kumar and Agarwal (2025)	Qualitative grounded theory	Category 1	Y/Y/Y/Y/Y
Caravelli et al. (2025)	Cross-sectional survey	Category 4	Y/N/Y/CT/Y
Sinlapanuntakul and Zachry (2025)	Mixed methods	Category 5	Qual: Y/Y/Y/Y/Y; Quant: Y/Y/Y/N/Y; Integration: Y/Y/Y/Y/Y
Javornik et al. (2022)	Sequential mixed methods	Category 5	Qual: Y/Y/Y/Y/Y; Quant: Y/CT/Y/CT/Y; Integration: Y/Y/Y/Y/Y
Javornik et al. (2021)	3 experiments + survey	Categories 2 and 4	Exp.: CT/Y/Y/N/Y; Survey: Y/CT/Y/CT/Y
Garg et al. (2026)	Two cross-sectional survey studies	Category 4	Y/CT/Y/CT/Y

Note. Y = Yes; N = No; CT = Can’t tell. MMAT criteria were applied according to the methodological design of each study. Category 1 = qualitative studies; Category 2 = randomized controlled trials; Category 3 = non-randomized studies; Category 4 = quantitative descriptive studies; Category 5 = mixed-methods studies. For mixed-methods studies, “Qual” refers to qualitative criteria 1.1–1.5, “Quant” refers to the applicable quantitative criteria, and “Integration” refers to mixed-methods criteria MM1–MM5. For Javornik et al. (2021), Studies 1–3 were assessed using Category 2 criteria and Study 4 using Category 4 criteria.

**Table 6:** Detailed MMAT criterion assessment of included studies.

Study	MMAT Criterion	Judgment	Basis for Judgment
Schroeder and Behm-Morawitz (2025)	C1. Appropriate randomization	CT	Random assignment was reported, but the randomization procedure was not described.
	C2. Groups comparable at baseline	CT	No explicit baseline comparison between experimental conditions was reported.
	C3. Complete outcome data	Y	Exclusions and the final analytic sample were clearly reported.
	C4. Blinded outcome assessment	N	Outcomes were self-reported and participants were aware of their condition.
	C5. Adherence to intervention	Y	Protocol adherence was monitored and participants using unauthorized filters were excluded.
Kumar and Agarwal (2025)	1.1 Appropriate qualitative approach	Y	Grounded theory was appropriate for the exploratory research questions.
	1.2 Adequate data collection	Y	Semi-structured interviews with probing were conducted until theoretical saturation.
	1.3 Findings adequately derived	Y	Open, axial and selective coding with expert validation was reported.
	1.4 Interpretation substantiated	Y	Verbatim participant quotations supported the findings.
	1.5 Coherence	Y	A clear path from interview data to coding and conceptual model was provided.
Caravelli et al. (2025)	4.1 Relevant sampling strategy	Y	Sampling directly targeted TikTok users relevant to the research question.
	4.2 Representative sample	N	Single-university, predominantly female and White undergraduate sample limited representativeness.
	4.3 Appropriate measurements	Y	Established and newly validated measures with reliability and factor analyses were reported.
	4.4 Low risk of nonresponse bias	CT	Response rate and responder/non-responder comparisons were not reported.
	4.5 Appropriate statistical analysis	Y	Regression analyses and assumption checks were appropriately reported.

Note. MMAT = Mixed Methods Appraisal Tool; Y = Yes; N = No; CT = Cannot Tell.

**Table 7:** MMAT assessment of randomized/experimental studies.

Study	C1 Randomization	C2 Baseline Comparability	C3 Complete Outcomes	C4 Blinding	C5 Adherence
Schroeder and Behm-Morawitz (2025)	CT	CT	Y	N	Y
Javornik et al. (2021), Studies 1–3	CT	Y	Y	N	Y

Note. CT = Can’t tell; Y = Yes; N = No. C1 = appropriate randomization; C2 = baseline comparability; C3 = complete outcome data; C4 = blinded outcome assessment; C5 = adherence to the intervention.

**Table 8:** MMAT assessment of qualitative components.

Study	1.1 Appropriate Approach	1.2 Data Collection	1.3 Findings Derived	1.4 Interpretation Substantiated	1.5 Coherence
Fiedler et al. (2025)	Y	Y	CT	CT	Y
Kumar and Agarwal (2025)	Y	Y	Y	Y	Y
Sinlapanuntakul and Zachry (2025)	Y	Y	Y	Y	Y
Javornik et al. (2022)	Y	Y	Y	Y	Y

Note. Y = Yes; CT = Can’t tell; 1.1 = appropriateness of the qualitative approach; 1.2 = adequacy of data collection methods; 1.3 = adequacy of derivation of findings; 1.4 = substantiation of interpretation by data; 1.5 = coherence between qualitative data sources, collection, analysis, and interpretation.

**Table 9:** MMAT assessment of quantitative descriptive components.

Study	4.1 Sampling Strategy	4.2 Representativeness	4.3 Measurements	4.4 Nonresponse Bias	4.5 Statistical Analysis
Caravelli et al. (2025)	Y	N	Y	CT	Y
Javornik et al. (2022), Study 6	Y	CT	Y	CT	Y
Javornik et al. (2021), Study 7	Y	CT	Y	CT	Y
Garg et al. (2026)	Y	CT	Y	CT	Y

Note. Y = Yes; N = No; CT = Can’t tell; 4.1 = relevance of the sampling strategy; 4.2 = representativeness of the sample; 4.3 = appropriateness of measurements; 4.4 = risk of nonresponse bias; 4.5 = appropriateness of statistical analysis.

**Table 10:** MMAT assessment of mixed-methods integration.

Study	MM1 Rationale	MM2 Integration	MM3 Integrated Interpretation	MM4 Divergences Addressed	MM5 Quality Criteria
Fiedler et al. (2025)	Y	CT	CT	CT	CT
Sinlapanuntakul and Zachry (2025)	Y	Y	Y	Y	Y
Javornik et al. (2022)	Y	Y	Y	Y	Y

Note. Y = Yes; CT = Can’t tell. MM1 = rationale for using a mixed-methods design; MM2 = integration of qualitative and quantitative components; MM3 = interpretation of integrated outputs; MM4 = addressing divergences between qualitative and quantitative findings; MM5 = adherence to the quality criteria of the respective methodological traditions.

## Data Availability

All data analyzed in this review were extracted from published scientific literature.

## Supplementary Materials

The following supporting information can be downloaded at: https://www.mdpi.com/article/10.3390/bs16101822/s1, Table S1: PRISMA 2020 Checklist.
