# Supplementary material for: When the AI Self Becomes the Ideal Self: A Systematic Review of AI-Enhanced Self-Discrepancy, Appearance Anxiety, and Protective Psychological Factors
Source: Behav Sci (Basel). 2026 Oct 5;16(10):1822. doi: 10.3390/bs16101822 (PMC13701967; doi:10.3390/bs16101822)
Supplement: Supplementary file 1 [file behavsci-16-01822-s001.zip › behavsci-4575451-supplementary.pdf]

PRISMA 2020 Checklist

Section and Topic	Item #	Checklist item	Location where item is reported
**TITLE**			
Title	1	Identify the report as a systematic review.	Title page - title includes "A Systematic Review"
**ABSTRACT**			
Abstract	2	See the PRISMA 2020 for Abstracts checklist.	Abstract
**INTRODUCTION**			
Rationale	3	Describe the rationale for the review in the context of existing knowledge.	Introduction, (Section 1.1 Research Gap).
Objectives	4	Provide an explicit statement of the objective(s) or question(s) the review addresses.	Introduction, final paragraph, (before Method).
**METHODS**			
Eligibility criteria	5	Specify the inclusion and exclusion criteria for the review and how studies were grouped for the syntheses.	Method, 2.3 Eligibility Criteria; Table 1 (Inclusion and Exclusion Criteria), Table 2 (Technological Sub-Types).
Information sources	6	Specify all databases, registers, websites, organisations, reference lists and other sources searched or consulted to identify studies. Specify the date when each source was last searched or consulted.	Method, 2.2 Sources of Information and Search Strategy-Scopus (searched by A.S., [June,2026]) and APA PsycInfo (searched by S.C., [September,2026]), records from January 2021–May 2026.
Search strategy	7	Present the full search strategies for all databases, registers and websites, including any filters and limits used.	Method, 2.2 Sources of Information and Search Strategy (full Boolean search string reported for Scopus; equivalent APA PsycInfo string in Supplementary Table S1).
Selection process	8	Specify the methods used to decide whether a study met the inclusion criteria of the review, including how many reviewers screened each record and each report retrieved, whether they worked independently, and if applicable, details of automation tools used in the process.	Method, 2.4 Study Selection, reports that screening, extraction, and quality appraisal were carried out by the first and second authors (A.S. and

Section and Topic	Item #	Checklist item	Location where item is reported
			N.M.); first-author screening at both title/abstract and full-text stages, with second-author independent verification of records/reports excluded at each stage; consensus resolution of any flagged disagreements; no automation tools used; no inter-rater agreement statistic recorded (see Limitations, Section 4.2). Records identified via APA PsycInfo by the third author (S.C.) were provided to the first author and screened using the identical procedure (Section 2.2).
Data collection process	9	Specify the methods used to collect data from reports, including how many reviewers collected data from each report, whether they worked independently, any processes for obtaining or confirming data from study investigators, and if applicable, details of automation tools used in the process.	Method, 2.5 Data Extraction,reports extraction by the first author, independent checking of all extracted data by the second author, review of the extraction form by both authors against two included studies prior to full extraction, and consensus resolution of discrepancies; no automation tools used.
Data items	10a	List and define all outcomes for which data were sought. Specify whether all results that were compatible with each outcome domain in each study were sought (e.g. for all measures, time points, analyses), and if not, the methods used to decide which results to collect.	Method, 2.5 Data Extraction (variables listed); Table 1 (Outcomes row), p. [10]

Section and Topic	Item #	Checklist item	Location where item is reported
	10b	List and define all other variables for which data were sought (e.g. participant and intervention characteristics, funding sources). Describe any assumptions made about any missing or unclear information.	Method, 2.5 Data Extraction (author, year, country, sample, design, technological context and sub-type, variables, theoretical frameworks invoked, findings, protective factors).
Study risk of bias assessment	11	Specify the methods used to assess risk of bias in the included studies, including details of the tool(s) used, how many reviewers assessed each study and whether they worked independently, and if applicable, details of automation tools used in the process.	Method, 2.6 Evaluation of Quality and 2.7 Risk of Bias Assessment (MMAT 2018), reports MMAT appraisal conducted by the first author, with the second author independently reviewing all criterion-level judgments against the source reports and discrepancies resolved by discussion; no automation tools used.
Effect measures	12	Specify for each outcome the effect measure(s) (e.g. risk ratio, mean difference) used in the synthesis or presentation of results.	Not reported - no meta-analysis/effect-measure synthesis was conducted (narrative thematic synthesis only).
Synthesis methods	13a	Describe the processes used to decide which studies were eligible for each synthesis (e.g. tabulating the study intervention characteristics and comparing against the planned groups for each synthesis (item #5)).	Method, 2.3 Eligibility Criteria and 2.4 Study Selection.
	13b	Describe any methods required to prepare the data for presentation or synthesis, such as handling of missing summary statistics, or data conversions.	Not explicitly reported — no data conversion/missing-data handling described.
	13c	Describe any methods used to tabulate or visually display results of individual studies and syntheses.	Table 4 (Characteristics of included studies); Table 3 (Protective and contextual constructs by

Section and Topic	Item #	Checklist item	Location where item is reported
			taxonomic category); Tables 5-6 (MMAT results).
	13d	Describe any methods used to synthesize results and provide a rationale for the choice(s). If meta-analysis was performed, describe the model(s), method(s) to identify the presence and extent of statistical heterogeneity, and software package(s) used.	Method, 2.8 Synthesis of Data (two-phase coding process — open coding independent of the three guiding theories, followed by theoretical organization; rationale for narrative thematic synthesis given heterogeneity).
	13e	Describe any methods used to explore possible causes of heterogeneity among study results (e.g. subgroup analysis, meta-regression).	Not applicable - no meta-analysis performed, so heterogeneity exploration methods not applicable
	13f	Describe any sensitivity analyses conducted to assess robustness of the synthesized results.	Not applicable - no sensitivity analyses conducted (narrative synthesis)
Reporting bias assessment	14	Describe any methods used to assess risk of bias due to missing results in a synthesis (arising from reporting biases).	Not reported - no formal reporting-bias assessment described.
Certainty assessment	15	Describe any methods used to assess certainty (or confidence) in the body of evidence for an outcome.	Not reported - no formal certainty/confidence grading (e.g., GRADE) was conducted.
**RESULTS**			
Study selection	16a	Describe the results of the search and selection process, from the number of records identified in the search to the number of studies included in the review, ideally using a flow diagram.	Results, Section 3 opening paragraph and Figure 1 (PRISMA 2020 flow diagram, updated with two-database totals: 624 records identified, 517 after deduplication, 61 assessed at full text, 8 included).

Section and Topic	Item #	Checklist item	Location where item is reported
	16b	Cite studies that might appear to meet the inclusion criteria, but which were excluded, and explain why they were excluded.	Partially reported — Results paragraph reports the six exclusion-reason categories and counts (no AI/AR self-representation n=24; wrong population/context n=9; wrong outcome n=7; wrong study type n=5; duplicate/overlapping n=4; other n=4) and references Figure 1; specific excluded studies not individually cited/explained in text.
Study characteristics	17	Cite each included study and present its characteristics.	Results, 3.1 Characteristics of the Included Studies; Table 4.
Risk of bias in studies	18	Present assessments of risk of bias for each included study.	Results, 3.2 Methodological Quality of Included Studies; Tables 5, 6, 7-10.
Results of individual studies	19	For all outcomes, present, for each study: (a) summary statistics for each group (where appropriate) and (b) an effect estimates and its precision (e.g. confidence/credible interval), ideally using structured tables or plots.	Results, 3.1.1-3.1.4 (narrative synthesis with reported statistics, e.g., β, p, d values per study); Table 4.
Results of syntheses	20a	For each synthesis, briefly summarise the characteristics and risk of bias among contributing studies.	Results, 3.1 and 3.2 (study characteristics and MMAT quality linked to each theme); Table 5.
	20b	Present results of all statistical syntheses conducted. If meta-analysis was done, present for each the summary estimate and its precision (e.g. confidence/credible interval) and measures of statistical heterogeneity. If comparing groups, describe the direction of the effect.	Results, 3.1.1-3.1.4 — narrative synthesis presented (no meta-analytic summary estimate, consistent with narrative thematic synthesis design),

Section and Topic	Item #	Checklist item	Location where item is reported
	20c	Present results of all investigations of possible causes of heterogeneity among study results.	Not applicable — no meta-analysis/heterogeneity statistics computed
	20d	Present results of all sensitivity analyses conducted to assess the robustness of the synthesized results.	Not applicable — no sensitivity analyses conducted
Reporting biases	21	Present assessments of risk of bias due to missing results (arising from reporting biases) for each synthesis assessed.	Not reported — no formal reporting-bias assessment presented.
Certainty of evidence	22	Present assessments of certainty (or confidence) in the body of evidence for each outcome assessed.	Not reported — no formal certainty-of-evidence grading presented
**DISCUSSION**			
Discussion	23a	Provide a general interpretation of the results in the context of other evidence.	Discussion, Section 4, opening paragraphs and subsections ("AI/AR representations as distinctive ideal selves," "Representation type... emerges as a potential boundary condition," "Protective and contextual factors...," "Positive body image: an identified gap...").
	23b	Discuss any limitations of the evidence included in the review.	Discussion, "Calibrating conclusions to evidence quality" paragraph; Section 4.2 Limitations.
	23c	Discuss any limitations of the review processes used.	Section 4.2 Limitations (two-database search with potential composition effects on the corpus, English-only, small k=8 corpus, single-screener-with-independent-verification model,

PRISMA

PRISMA 2020 Checklist

Section and Topic	Item #	Checklist item	Location where item is reported
			narrative vs meta-analytic synthesis).
	23d	Discuss implications of the results for practice, policy, and future research.	Section 4.1 Implications.
**OTHER INFORMATION**			
Registration and protocol	24a	Provide registration information for the review, including register name and registration number, or state that the review was not registered.	Method, 2.1 Review Design ("This review was not registered.").
	24b	Indicate where the review protocol can be accessed, or state that a protocol was not prepared.	Not reported — no protocol availability statement included.
	24c	Describe and explain any amendments to information provided at registration or in the protocol.	Not applicable — no registration/protocol amendments (review not registered).
Support	25	Describe sources of financial or non-financial support for the review, and the role of the funders or sponsors in the review.	Statements and Declarations, Funding ("No funding was received for conducting this research").
Competing interests	26	Declare any competing interests of review authors.	Statements and Declarations, Competing interests ("None").
Availability of data, code and other materials	27	Report which of the following are publicly available and where they can be found: template data collection forms; data extracted from included studies; data used for all analyses; analytic code; any other materials used in the review.	Statements and Declarations, Data Availability ("All data analyzed in this review was extracted from published scientific literature.").

From: Page MJ, McKenzie JE, Bossuyt PM, Boutron I, Hoffmann TC, Mulrow CD, et al. The PRISMA 2020 statement: an updated guideline for reporting systematic reviews. BMJ 2021;372:n71. doi: 10.1136/bmj.n71. This work is licensed under CC BY 4.0. To view a copy of this license, visit https://creativecommons.org/licenses/by/4.0/
